# Development of a Questionnaire to Assess Knowledge and Perceptions about Edible Insects

**DOI:** 10.3390/insects13010047

**Published:** 2021-12-31

**Authors:** Raquel P. F. Guiné, Sofia G. Florença, Cristina A. Costa, Paula M. R. Correia, Manuela Ferreira, João Duarte, Ana P. Cardoso, Sofia Campos, Ofélia Anjos

**Affiliations:** 1 CERNAS—Research Centre for Natural Resources, Environment and Society, Polytechnic Institute of Viseu, 3504-510 Viseu, Portugal; amarocosta@sc.ipv.pt (C.A.C.); paulacorreia@esav.ipv.pt (P.M.R.C.); 2 Faculty of Food and Nutrition Sciences, University of Porto, 4200-465 Porto, Portugal; sofiaflorenca@outlook.com; 3 Health Sciences Research Unit: Nursing (UICISA: E), Polytechnic Institute of Viseu, 3504-510 Viseu, Portugal; mmcferreira@gmail.com (M.F.); duarte.johnny@gmail.com (J.D.); 4 CIDEI—Centre for Studies in Education and Innovation, Polytechnic Institute of Viseu, 3504-510 Viseu, Portugal; a.p.cardoso@esev.ipv.pt (A.P.C.); sofiamargaridacampos@gmail.com (S.C.); 5 School of Agriculture, Polytechnic Institute of Castelo Branco, 6001-909 Castelo Branco, Portugal; ofelia@ipcb.pt; 6 Forest Research Centre, School of Agriculture, University of Lisbon, 1349-017 Lisbon, Portugal; 7 Centro de Biotecnologia de Plantas da Beira Interior, 6001-909 Castelo Branco, Portugal

**Keywords:** instrument validation, scale, questionnaire survey, edible insects consumption

## Abstract

**Simple Summary:**

Edible insects are considered a traditional food in many countries, especially in Asia, Africa and South America, but in other countries, for example, in Europe, they are not readily accepted and people tend to have some disgust towards this type of food. Lately, however, edible insects have been pointed to as a possibly more sustainable source of animal protein, allied with other nutritional and environmental advantages. In this way, they can be considered as a future food that could help mitigate hunger and malnutrition. Additionally, new gastronomic trends are already targeting this area for exploring new potentialities. The objective of this work was to develop and validate a questionnaire to assess consumers’ perceptions of and knowledge about edible insects, focusing on different perspectives, such as cultural influences, gastronomic potential, the sustainability of food systems, economic and commercialization aspects or nutrition and health. The validation of this questionnaire confirms its usefulness for investigating consumer perceptions of and knowledge about edible insects, making possible its application in different countries. As a result, actions could be planned to improve the acceptability of edible insects in societies unaccustomed to this type of food, maybe by benefiting from the experience of countries wherein insects are considered valuable foods.

**Abstract:**

Edible insects (EI) have been consumed as traditional foods in many parts of the globe, but in other regions, they are not readily accepted, particularly in Western countries. However, because EI are suggested to constitute a more sustainable protein food as compared with other sources of animal protein, they can be considered a future food that could help mitigate hunger and malnutrition. Additionally, new gastronomic trends are already targeting this area for exploring new potentialities. The objective of this work was to develop and validate a questionnaire to assess consumers’ perceptions and knowledge about EI in seven different domains: D1. Culture and Tradition, D2. Gastronomic Innovation and Gourmet Kitchen, D3. Environment and Sustainability, D4. Economic and Social Aspects, D5. Commercialization and Marketing, D6. Nutritional Aspects and D7. Health Effects. The 64 items were subjected to item analysis and reliability analysis for validation, and factor analysis was also conducted to identify a grouping structure. The results validated all the items of the seven subscales with high values of Cronbach’s alpha (α = 0.732 for D1, α = 0.795 for D2, α = 0.882 for D3, α = 0.742 for D4, α = 0.675 for D5, α = 0.799 for D6 and α = 0.788 for D7). However, by eliminating 17 items, the final values of the alpha increased in all subscales. Factor analysis with extraction by principal component analysis with varimax rotation extracted 14 factors that explained, in total, 65% of the variance, although the first two factors were the most important (35.7% variance explained). In conclusion, the confirmed usefulness of the questionnaire has been hereby validated for assessing consumer perceptions of and knowledge about EI.

## 1. Introduction

The Sustainable Development Goals (SDGs) were established by the General Assembly of the United Nations (UN) in 2015, intended to work towards a sustainable society seeking economic prosperity while comprising social and environmental concerns. Climate change and the SDGs constantly remind one of the growing interest in achieving food and nutrition security, most especially goals 2—Erase Hunger, 3—Establish Good Health and Well-Being, 14—Develop Life Below Water and 15—Advance Life On Land. Although great efforts have been carried out in the last decades by developing strategies and policies towards achieving global food security, it is a fact that still, today, approximately 10% of the worldwide population suffers from severe levels of food insecurity [1,2].

In Western countries, it is common to consume considerably high amounts of protein, much higher when compared with such consumption in developing countries. It is also a fact that the highest proportion of protein consumed comes from animal sources rather than vegetable sources. It is expected that the demand for meat products might duplicate within a few decades, on one hand, due to population growth and, on the other hand, because in some developed and developing countries, incomes are increasing [3]. Livestock production has been reported as having a huge impact on the loss of biodiversity, resulting in an impoverishment of the ecosystems; in the reduction of available freshwater, which is essential to support all forms of life on Earth; and causing climate change, particularly, contributing to the global warming caused by greenhouse gases (GHG), among others [4]. The impact of world beef production is immense; since this continues to expand, much of which owing to the devastation of natural ecosystems as a way to obtain pastures for cattle, and this being particularly problematic in South America, with the destruction of tropical rain forests that are considered part of the planet’s lungs. This forest devastation to establish pastures is precipitously interfering with the ecosystem’s functioning [5]. However, there is no agreement whatsoever on who is to blame when it comes to the impact of the agrifood systems and food supply chains on the environment. Some consider that livestock is highly responsible for the increase in GHG emissions, while others refute those accusations by saying that range livestock production is much more efficient in terms of environmental impact and energy expenditure when compared with other systems of food production based on land, including the vegetable ones and especially as concerns some extensive crops [6].

It has been estimated that the regular consumption of insects has been part of the traditional diets of over two billion people worldwide [7]. Insects have been identified as a more sustainable alternative when compared with other, more conventional animal protein sources [6,8,9,10]. In this way, they can contribute to greatly relieving the pressure on the planet and on ecosystems given the imminent need to feed the world population, which is constantly growing. It has been stated that the consumption of edible insects (EI) as a non-conventional source of animal protein or even as a complementary meat substitute can in fact, present several advantages. One of them is related to nutritional composition, since it has been described that many EI possess unique nutritive properties. Additionally, EI seem to provide a means of ingesting bioactive compounds with proven beneficial health effects [11,12,13,14,15]. It is also important that insect production has a considerably lower impact on the environment, as compared with other sources of animal protein, which feature lower emissions of GHG, less space required for insect farming, lower energy consumption and a reduced need of freshwater. Finally, insects can constitute an opportunity for economic growth, local and regional development and the livelihood of poor families, providing income [3,16,17,18].

Many chefs have also joined a trend of using insects in their culinary preparations, bringing insects to the top gastronomy level [19]. In particular, they highlight their organoleptic qualities combined with a recognized nutritional value, evidenced by scientific studies. Dion-Poulin [19] studied the acceptability of insect ingredients by innovative student chefs and concluded that understanding the perceptions of innovative chefs about the use of insect-based foods can contribute to the promotion and wider use of EI in gastronomy, and eventually improve their acceptability by consumers. However, in some markets, insects or insect-based products are not readily accepted due to some degree of food neophobia [20]. In this way, it becomes relevant to know in what way the sociocultural identity, the level of knowledge and awareness for sustainability issues contributes to the acceptance or rejection of this type of food. The EISuFood Project aims to study eating habits, knowledge and perceptions of consumers towards EI or their derived products. This work describes the pre-validation of a questionnaire developed in the ambit of the EISuFood project led by the CERNAS Research Centre (Polytechnic Institute of Viseu, Viseu, Portugal).

## 2. Materials and Methods

### 2.1. Instrument

The questionnaire used in this study was prepared to assess the perceptions and knowledge of citizens about EI, to be used, as previously mentioned, in the EISuFood project. The questions were formulated according to seven dimensions and based on the bibliographic reported information, as shown in Table A1 (Appendix A). To begin with, the draft version of the questionnaire was prepared in English by the members of the Portuguese team, who refined it and sent it to all partners of the project for evaluation and improvement. In total, 69 researchers from the 18 different aforementioned countries participated in the definition of the final version of the questionnaire. This version, resulting from the different contributions, was prepared on the basis of a discussion meeting among the Portuguese members of the team, thus resulting in a working version that was after translated from English to Portuguese. In the next step, this working version of the questionnaire was sent to a group of professionals of different areas for the first stage of semantic validation [21]—in the Portuguese language: food science (3), nutrition (1), agriculture (1), health sciences (1), psychology (1), educational sciences (1) and statistics (1). The working version was submitted to a pre-test, by application to 50 persons selected randomly among the Portuguese population, to detect possible misunderstandings and other problems of interpretation by the respondents, with the objective of correcting the questionnaire accordingly. Finally, the questionnaire was applied to a sample of at least 200 people to undertake statistical validation [22].

The draft version of the questionnaire contained, initially, 70 items, but after group discussion, 77 items were suggested resulting from all countries’ input. Yet, another refinement allowed restricting the number of items, either because some of them were very similar to others already in the questionnaire or because they were found redundant for the purpose on the study. Hence, a reasonable number of items was also selected to facilitate the data collection and avoid exhaustion of the participants. In total, 64 items were included in the final version of the questionnaire, as described in detail in Appendix A, which are, in summary, grouped in seven dimensions: D1. Culture and Tradition (10 items); D2. Gastronomic Innovation and Gourmet Kitchen (9 items); D3. Environment and Sustainability (11 items); D4. Economic and Social Aspects (6 items); D5. Commercialization and Marketing (8 items); D6. Nutritional Aspects (10 items); D7. Health Effects (10 items). All items were evaluated through a five-point Likert scale as follows: one = strongly disagree, two = disagree, three = no opinion, four = agree, five = strongly agree [23]. Additionally, the questionnaire included a section aimed at collecting data destined to sociodemographically characterize the sample.

### 2.2. Collection of Data for Pre-Test and Validation Test

This descriptive cross-sectional study was carried out using a non-probabilistic sample with 367 Portuguese participants. The questionnaires were distributed, after informed consent was obtained, only to adults (aged 18 or over). All ethical issues were strictly observed when formulating and administering the questionnaire, which was approved by the Ethics Committee with reference 45/SUB/2021. The data collection started in July 2021 and continued until November 2021. The questionnaire was delivered during COVID-19 restrictions, so the electronic platform Google Forms was used to deliver the questionnaire, and recruitment was done by email and social media, complemented with a direct interview, particularly in the early stages of the pre-test. The objective of this phase was to verify if the questions were perceptible and if they had the inherent qualities to measure what was initially outlined, i.e., perceptions and knowledge about EI [22]. Hence, the pre-test consisted in administering the questionnaire by direct interview to a small sample, which, according to Hill and Hill [22], should be composed of 50 participants. This pre-test allowed identifying some questions that were eventually found not very clear to participants, and, therefore, were rewritten and/or reformulated accordingly.

This modified version of the questionnaire was applied to a sample of 367 participants, which was higher than the minimum number of participants required for validation advised by Hill and Hill [22], of 100. On the other hand, to ensure practical validity, the number of participants should be five or six times that of the number of items in the questionnaire; in this case, corresponding to a minimum of 325 participants, because the final version of the questionnaire contained 64 items.

### 2.3. Sample Characterization

The sample was constitutive of 367 Portuguese participants, of which one third were men and two-thirds were women (22.9% and 76.6%, respectively). The asymmetry between men and women was not intended and resulted from the data collection, since more women chose voluntarily to participate in the survey than men, even though the questionnaire was disclosed equally to members of both sexes. This asymmetry has been observed in plenty of other studies using convenience samples recruited on the internet, as in the present case [24,25,26,27].

Regarding education level, 36.2% had a post-graduate education, 18.0% had completed a university degree and 45.8% had not completed a university degree. Regarding living environment, 32.7% live in rural areas, 56.9% in urban areas and 10.4% in suburban areas. With respect to age, it varied between 18 and 85 years, with an average of 32.49 ± 15.53 years, with men being older than women, on average (37.74 ± 15.74 and 31.01 ± 15.17 years, respectively).

### 2.4. Statistical Analyses

**Figure 1:**
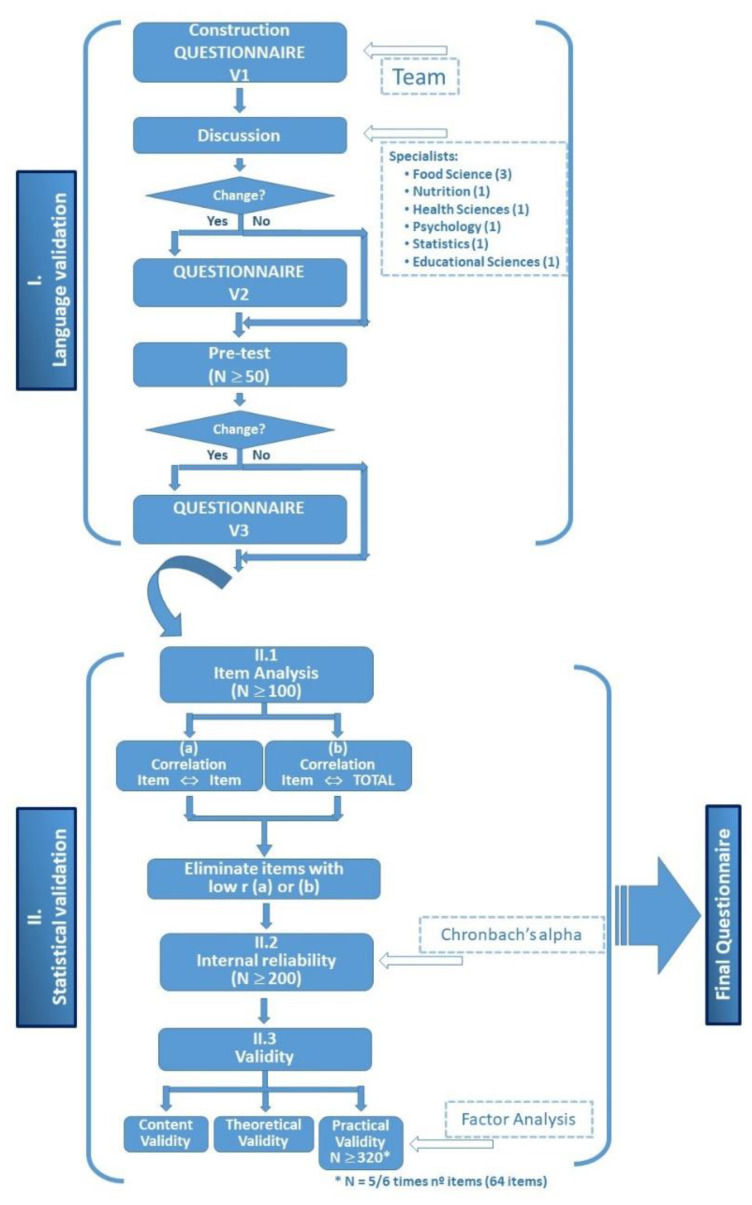
Validation algorithm.

After linguistic validation, the statistical validation of the questionnaire was achieved, following the procedure described in Figure 1.

Basic descriptive statistical tools were used for the exploratory analysis of the data. Additionally, item analysis was performed on two levels: item–item correlations and item–total correlations. Item analysis can be applied to samples of over 100 participants [22]. To perform item analysis, Pearson correlation coefficients were calculated, which measure the association between two variables according to the magnitude of the absolute value [28,29,30]. If 0.00 < r < 0.10 the association is very weak, if 0.10 ≤ r < 0.30 the association is weak, if 0.30 ≤ r < 0.50 the association is moderate, if 0.50 ≤ r < 0.70 the association is strong and if 0.70 ≤ r < 1.00 the association is very strong. For r = 0 there is no association, and for r = 1 the association is perfect.

The reliability of the scales for each of the seven independent dimensions considered was evaluated through the calculation of the Cronbach’s alpha (α), which measures the internal consistency of the different statements evaluated within a certain group [31]. The values of Cronbach’s alpha (α) range from 0.0 to 1.0. Higher scores indicate a more reliable, homogenous scale in which the individual items in each domain of the questionnaire reliably measure the domain core concept [32]. According to Hill and Hill [22], the alpha can be interpreted as follows: α < 0.6—unacceptable internal reliability; 0.60 ≤ α < 0.70—weak internal reliability, 0.70 ≤ α < 0.80—acceptable internal reliability, 0.80 ≤ α < 0.90—good internal reliability and α ≥ 0.90—excellent internal reliability.

In a complementary stage of the data analysis, factor analysis (FA) was also used, considering all 64 items. Firstly, the suitability of the data for this kind of analysis was tested through evaluation of the correlation matrix and the values of MSA (measure of sampling adequacy) in the anti-image matrix, the Kaiser–Meyer–Olkin measure of adequacy of the sample (KMO) and Bartlett’s test [24,33]. The solution was obtained through extraction with the principal component analysis (PCA) method with varimax rotation. The Kaiser criterion was used to stipulate the number of components to retain, which means considering eigenvalues ≥1. Communalities indicated the percentage of variance explained (VE) by the factors extracted [31], and they were to be equal to 0.5 or higher [30,34]. To determine the internal consistency in each factor, we again used Cronbach’s alpha (α) [31,35].

The analysis of the data used SPSS software from IBM Inc. (version 26, Armonk, NY, USA).

## 3. Results

### 3.1. Internal Structure Validation

The questionnaire structure and the seven subscales were validated by item analysis, including item–item and item–total correlations, as well as reliability through the calculation of Cronbach’s alpha, the results of which are presented in the following sections.

#### 3.1.1. Item Analysis

Table 1 shows the values of the Pearson correlations for all subscales considered. With respect to the correlations between the items in subscale D1—Culture and Tradition, and between each item and the total value (corresponding to the sum of all items in this subscale), the highest values of item–item correlations are 0.418 (item 10 vs. 5) and 0.405 (item 8 vs. 7), which are moderate in strength but significant in both cases (*p* < 0.01). On the other hand, the highest values for the item–total correlations are 0.445, 0.441 and 0.417, which are also moderate and significant at the level of significance of 1%, and correspond to items 9, 10 and 1, respectively.

The correlations in Table 1 for subscale D2—Gastronomic Innovation and Gourmet Kitchen are comparatively higher than for the previous case, with two item–item correlations above 0.7 (for item 7 vs. 6 r = 0.748 and for item 6 vs. 5 r = 0.725), so corresponding to very strong correlations and both are significant at the 0.01 level. There are eight strong correlations (r between 0.5 and 0.7), all significant. As concerns the item–total correlations, only one is lower than 0.5 (item 3 vs. total), there are three strong (for items 1, 2 and 4) and five are very strong (for items 5, 6, 7, 8 and 9). The strongest item–total correlation is for item 7, with a value of r = 0.798.

For the item analysis of subscale D3—Environment and Sustainability, Table 1 shows again a high number of strong (r between 0.5 and 0.7) or very strong item–item correlations (r > 0.7), and all significant at 0.01 level, being the highest value r = 0.724 (item 5 vs. 1). The weakest items appear to be 6, 9 and 10. In what concerns the correlations item–total, seven out of eleven are very strong, with the highest value r = 0.810 for item 5.

The item analysis for subscale D4—Economic and Social Aspects (Table 1) reveals five strong correlations (r between 0.5 and 0.7) and one very strong correlation (r = 0.706 for item 2 vs. 1), in all cases significant at the 0.01 level. With regards to the item–total correlations, five are very strong, the highest value being r = 0.801, for item 5.

Table 1 also shows the Pearson correlations from the item analysis of subscale D5—Commercialization and Marketing. In this case, the number of strong correlations is lower, and there are only two item–total correlations higher than 0.7, for items 6 and 7. The highest item–item correlation was r = 0.551, for item 7 vs. 6. Although not so high, these correlations are nonetheless significant at the 0.01 level.

With respect to subscale D6—Nutritional Aspects, the item–item correlations in Table 1 reveal four very strong (over 0.7) and eleven strong correlations (r between 0.5 and 0.7. The item–total correlations are in general strong or very strong, with the highest value belonging to item 6 (r = 0.840), closely followed by item 8 (r = 0.802); both correlations are significant at the 0.01 level.

Finally, for the last subscale, D7—Health Effects, the item–item correlations are not so high as in the other subscales, with only seven strong correlations and none very strong, although of the item–total correlations, two very strong correlations were found for items 2 and 7 (0.703 and 0.705, respectively), as were six strong correlations (items 1, 4, 6, 8, 9 and 10).

#### 3.1.2. Internal Reliability

The internal reliability for all seven subscales is shown in Table 2. Considering the subscale D1 (Culture and Tradition), the internal reliability, including all ten items, revealed a global Cronbach’s alpha value of 0.732, which is acceptable [22], but this could be improved by eliminating item number 2, and therefore the final value for this scale was α = 0.740, still in the range of acceptable values for alpha. With respect to subscale D2 (Gastronomic Innovation and Gourmet Kitchen), the value of alpha considering the nine original items was acceptable (0.795), but it was very much improved by the removal of some items, specifically 1, 2, 3, and 4, thus yielding a very high value of alpha (α = 0.901), which indicates excellent internal reliability. For subscale D3 (Environment and Sustainability), the whole set of eleven items had good reliability (α = 0.882), but this was further improved by removing items 6, 8, 9, and 10, thus yielding an excellent internal reliability (α = 0.932) again. The subscale D4 (Economic and Social Aspects), considering the initial six items had acceptable reliability (α = 0.742), was again improved by removing one item (item 3), and the final value of alpha was increased to good (α = 0.843). Subscale D5 (Commercialization and Marketing), with its original eight items, had a weak alpha value (α = 0.675), but the removal of items 2 and 8 improved the internal reliability of the subscale to acceptable, with a value very close to 0.8 (α = 0.793). The subscale D6 (Nutritional Aspects) originally had ten items, and the corresponding alpha was acceptable (α = 0.799), which was, however, increased to excellent (α = 0.912) by removing some items (1, 4, 9 and 10). As concerns the last subscale D7 (Health Effects), the internal reliability of the original set of ten items was acceptable (α = 0.788), but this was increased to good (α = 0.832) by removing items 3 and 5. These results indicate that all subscales had acceptable internal reliability in their original forms, but still could be improved by removing some key items in each case.

### 3.2. Factor Analysis

The 64 items considered by joining the seven subscales were submitted to FA, which was revealed to be a statistical technique suitable for the present data because the results of the Bartlett’s test ensured a highly significant *p*-value (*p* < 0.0005). This result lead to the rejection of the null hypothesis H0, according to which “The correlation matrix is equal to the identity matrix”, meaning that there are important correlations between the variables, and this was previously confirmed through item analysis. Additionally, the value of the KMO measure of adequacy was excellent (0.915) according to the classification proposed by Kaiser and Rice [36]. The MSA values of the anti-image matrix (Table 3) were all higher than 0.5 (varying between 0.659 for item D3.8 and 0.966 for item D3.3), and this confirms that all the variables should be included in the analysis.

The rotated solution required sixteen iterations to converge and extracted fourteen factors, explaining 65.2% of the total variance (F1—28.0%, F2—7.7%, F3—4.1%, F4—3.7%, F5—3.4%, with all other factors explaining less than 3% each). The communalities revealed that the variable that had the highest fraction of its variance explained by the solution was item 11 in the D3 group (VE = 80.5%), and the lowest was for item 1 in group D2 (VE = 44.2%). Table A2 (Appendix B) presents the results of FA, i.e., the factors and the contributing variables with their corresponding loadings. Factor F1 included essentially items from groups D3 (Environment and Sustainability) and D4 (Economic and Social Aspects), factor F2 was mostly linked to items in D6 (Nutritional Aspects), factor F3 was linked with items from group D2 (Gastronomic Innovation and Gourmet Kitchen), F4 and F5 were mostly associated with items in D7 (Health Effects), F6 was linked with items in D5 (Commercialization and Marketing), and factors F8 and F9 were both equally linked with items from D1 group (Culture and Tradition). The highest factor loading was 0.816, for item D2.6 to factor F3 (Table A2 in Appendix B).

The solution was validated through the Cronbach’s alpha (α), which measures the internal consistency within each of the factors [31]. The values of Cronbach’s alpha for the initial group of items in factors F1 and F2 were higher than 0.9 (Table 4), which can be classified as excellent [37,38,39]. However, they could still be improved by removing some variables. Hence, factors F1, F2 and F, in the final structure, all had alpha values considered excellent (0.939, 0.912 and 0.901, respectively). With respect to factor F4, the value of alpha could not be improved, and the final group of items remained at seven (α = 0.827, is good). The alpha values in Table 4 show that factors F5, F6, F7 and F10 had acceptable values of alpha (higher than 0.7). On the other hand, the remaining factors had values of alpha that were weak or unacceptable.

## 4. Discussion

This work describes the validation of a scale to measure knowledge and perceptions about EI, which are pointed out as a source of animal protein alternative to traditional livestock production, producing considerable environmental advantages [10]. Insects are presented as a promising alternative food source that could lessen the environmental impact associated with meat production in Western cultures, this being so because insect production has a lower ecological footprint—it produces lower GHG emissions and requires less feed and water than conventional cattle [6]. It has been reported that insect farming is more environmentally friendly, due to the lower emissions of GHG, most especially when compared with the farming of cows, and also due to the lesser feed and water needed for insects and by being able to produce large quantities of insects in very small areas, avoiding the need of pastures. Additionally, insect farming represents a considerably lower economic investment for the producers and provides good income due to their higher efficiency in the conversion of rations when compared with other livestock [40]. For example, it has been reported that, for some insect species such as crickets or mealworms, only 40 L of water is needed to produce 1 kg of insect protein [41]. Finally, insects reproduce quickly and can be grown in different parts of the world [42]. These advantages have led the UN and the Food and Agricultural Organization (FAO) to suggest insects as a potential solution to mitigating the worldwide problem of hunger and malnutrition, patent in the food insecurity that is expected due to the rise in the world population [43].

As EI consumption is very natural and frequent in some cultures, but not at all in others—mostly in Western countries. It is important to assess people’s perceptions and knowledge about EI as food [44,45,46]. To carry out that objective, it is imperative to have an instrument that might be used, after some validation, to ensure that the correct information is being collected [21,35,47]. Although there are, in the literature, some instruments related to this topic, such as, for example, the Food Neophobia Scale [48] which is the most used, it is true that it does not apply specifically to EI and does not cover the range of domains that were included in our questionnaire. This work describes the validation of the latter for future use. The scale, composed of 64 items distributed in the seven domains considered, is hereby validated, according to the results of the internal reliability analysis of all subscales, since the lowest value of alpha was for subscale D5, with all its eight items, but was still acceptable. However, it was further possible to discover that, of the items considered, some were less strong, and their elimination from the scales allowed improving internal reliability. As those items were those that might eventually be more problematic, the discussion will focus more specifically on them.

The dimension Culture and Tradition was designed to measure, for different samples, to what extent EI are or are not part of their cultural heritage [49]. EI collected from the wild are frequently consumed either as a main course or as snack food by people in various rural communities in many parts of the world, including Southeast Asia, the Pacific, sub-Saharan Africa and Central and South America [49]. However, entomophagy is not readily accepted in most western countries [50]. Insect consumption is described as closely associated with cultural values, religious festivities, local customs, taboos and traditional knowledge [50,51,52]. In this subscale, there was only one item not so strongly connected with the construct, which was D1.2 “Insects are considered a traditional food in my country”. In Portugal, in fact, EI are not at all traditional, and the diets tend to align with the Mediterranean diet (MD) or Westernized diets. Portugal was one of the countries that first subscribed to the application of the MD to the Intangible Cultural Heritage of the United Nations [53,54]. However, that dietary pattern has been changed to become aligned with less healthy dietary patterns. Sousa [55] reported low adherence to MD in Portugal, with a high impact on cardiovascular diseases. Although edible insects are not a traditional food in Portugal, studies have demonstrated that people are to some extent prone to start consuming products that contain edible insects as a complement to their diet, mostly motivated by their sustainability aspects [8,20]. This confirms that people are aligned with the need to promote more sustainable food supply chains in order to meet the goals established under the SDG of the UN.

The second dimension considered, Gastronomic Innovation and Gourmet Kitchen, is very relevant from the point of view of incentivizing and influencing power of certain key subjects to help improve the acceptability of EI. Improving the image of EI and increasing consumer acceptance are great challenges [9,56]. In this subscale, four items were identified as possibly more problematic: D2.1 “Insects are considered as exotic foods”, D2.2 “Insects are traded as treats/delicacies”, D2.3 “3. Insects are not suitable for human consumption” and D2.4 “Insects are associated with taboos and food neophobia”. In fact, these perceptions can be highly variable according to the cultural environment of the respondents [57,58]. The perception that they are not suitable for human consumption is a problem identified in many studies, and their safety has been an object of concern [59]. While Murefu et al. [60] focus on the safety aspects related to insects collected from the wild, Baiano [61] address the safety of reared insects, and Yates-Doerr [62] discuss the One Health biosecurity of EI. To this point, is it paramount to have appropriate regulations that protect the consumer and allow only the commercialization of safe insect-based foods. The European Market, in particular, is highly regulated and only very recently approved the second edible insect (*Locusta migratoria*). The Novel Food Regulation helps food businesses bring innovative foods to the European Union (EU) market, while guaranteeing their safety [63].

The dimension Environment and Sustainability is one of the aspects that could help consumers to shift into the adoption of EI, since modern consumers are more alert to these matters and are more prone to change their diets toward more sustainable food choices [64,65]. In this subscale, again, four items were identified as eventually less representative of the construct: D3.6 “The production of chicken protein requires much less water than insect protein”, D3.8 “The production of insect protein requires much more area than pig protein”, D3.9 “Insects are collected as a means of pest control for some cultivated crops” and D3.10 “Loss of biodiversity is lower with insect production compared with other animal food production”. All these refer to questions about knowledge and refer to aspects to which the respondents tended to vary widely in their way of responding. Additionally, two of them were given as false statements (D3.6 and D3.8), which may have contributed to the respondent’s difficulty. It is estimated that the production of insect protein requires less about half of the amount of the feed and water when compared with chicken protein, and about one fourth the area needed when compared with pig protein [6,20]. However, these facts are not known by the general population. It is also not known by the general public that insects are collected as a means of pest control for some cultivated crops, thus having a double advantage, helping to maintain the vigour of vegetable crops while providing a food source [49]. One other aspect that needs to be further communicated to the public is the role of insect farming in maintaining high biodiversity levels [49].

Regarding the dimension Economic and Social Aspects, of its five items, only one raised concern, D4.3 “The market of edible insects is expected to decline in the future”. In fact, it is reported that there will be an increasing trend in the market for EI, being expected to triple from about $400 million in 2018 to almost $1.2 billion in 2023, or even reach $8 billion by 2030 [6,66,67]. At present Asia–Pacific and Latin America markets account for about two-thirds of the EI market, but markets in Europe, North America, the Middle East and Africa are expected to increase [6]. One other dimension very closely related with this is Commercialization and Marketing, which revealed two less-strong items to build the construct: D5.2 “Edible insects are easy to find on sale in supermarkets” and D5.8 “Insect consumption is independent of marketing campaigns”. Portugal is a country without a tradition of consuming EI, and besides, the commercialization of this type of new food is highly limited by European regulations. Therefore, it is not yet easy to find this type of product for sale in supermarkets, the internet being the more accessible means of purchasing EI-based food products [20]. One of the biggest food supply chains operating in Portugal, the “Continente”, was the first to sell EI-based foods, having started very recently, in August 2021 [68], because only in June 2021 had the Directorate General for Food and Veterinary Medicine (DGAV—Direção Geral da Alimentação e Veterinária) authorized the consumption of some species of insects in the country: *Acheta domesticus*, *Alphitobius diaperinus*, *Apis mellifera*, *Gryllodes sigillatus*, *Locusta migratória*, *Tenebrio mollitor* [69].

With respect to the dimension Nutritional Aspects, four items were identified as less strongly associated with the whole subscale: D6.1 “Insects have poor nutritional value”, D6.4 “Insect proteins are of poorer quality compared with other animal species”, D6.9 “Insects contain fat, including unsaturated fatty acids” and D6.10 “Insects contain anti-nutrients, such as oxalates and phytic acid”. The first two statements, D6.1 and D6.4, were given as false statements, and the respondents may have had trouble identifying them as false; on the other hand, they might only have considered that insects are not good food, by prejudice or by misinformation. Insects have a high nutritional value [11], providing, in general, high amounts of proteins, fats, vitamins and minerals. As an example, 100 g of caterpillars provide 76% of the recommended daily intake of protein and nearly 100% that of vitamins for humans [70]. However, they can also contain some anti-nutrients, like oxalates and phytic acid [60,71,72,73]. These anti-nutrients are compounds whose action within the human body reduces the bioavailability and/or utilization of nutrients if consumed in large quantities and over a long period of time [72].

The last dimension considered was Health Effects, with only two out of the ten items showing a lower consistency: D7.3 “Eating insects poses a substantial risk to human health” and D7.5 “Insects and insect-based foods are often infected by pathogens and parasites”. These items are essentially related to the risks associated with the consumption of EI. Consumers tend to be suspicious about those foods they are not familiar with and consider them to pose a higher level of risk than other foods, especially with higher risks involved, such as with shellfish, for example. Hwang and Choe [56] used perceived risk theory to enhance the image of EI and concluded that this image influenced the behavioural intentions of consumers. Baker et al. [74] used information processing theory as well as risk perception theory to study consumers’ negative perceptions toward edible insect food products, and to find a means of decreasing those negative impressions, thus, facilitating the adoption of these foods. Although the perception of risks was high, the consumption of EI is safe, provided that all good practices are followed in their production and transformation, just as has happened with other types of food. Additionally, EI have been reported as having bioactive compounds with beneficial health effects in several studies [11,12,13,14,15].

Among European Countries, some limited research has been conducted through questionnaire surveys about the consumption of EI, and some recent works related to the consumption of insects among Europeans highlight important conclusions. For example, in Finland, a survey conducted by Niva and Vainio [75] suggests that consumers are motivated to shift to more sustainable diets, with 24% admitting an intention to increase their use of insect-based food products in the future. A study among Danish consumers [76] allowed developing a scale to predict the intention to consume insects, based on the measurement of attitudes towards entomophagy. In Germany, a quantitative investigation focusing on the willingness to consume insects [77] showed a significant effect of food neophobia and that sustainability issues would not act as a significant predictor of the intention to eat insects. Dupont and Fiebelkorn [78] reported that, for German children and adolescents, food disgust does not seem to influence the acceptance of insects as alternatives to meat. In a similar study conducted in Denmark [79] children demonstrated some neophobia towards the consumption of insect-based foods.

## 5. Conclusions

This work resulted in a validated instrument, designed to assess the knowledge about and perceptions of EI. The questionnaire is composed of 64 items, grouped into seven dimensions, all validated through item analysis and internal reliability by calculating the values of Cronbach’s alpha. Although the original version of the questionnaire was validated with 64 items, it was further observed that a final selection of 47 items would provide higher consistency in the different subscales considered. The factor analysis showed ten factors explaining, globally, about 65% of the total variance, but the first two were the most important, accounting for only 35.7% of the variance explained. The items that most contributed to the explained variance in factor F1 were essentially related to sustainability: D3.11, concerning the lower energy input in insect production; D3.2, concerning lower GHG emissions from insects relative to cows; and D3.4, concerning the lower need of feed for insects. Regarding the factor F2, the items most relevant to it were related to the nutritive value of insects: D6.5, concerning insects providing essential amino acids; D6.6, concerning vitamins of the B-group; and D6.7, concerning dietary fibre. In this way, given this pre-validation, it is possible to use the questionnaire for application in the ambit of the EISuFood project.

## Figures and Tables

**Table 1:** Item analysis to the seven subscales (Pearson correlations item–item and item–total).

D1—Culture and Tradition	**Items**	**1**	**2**	**3**	**4**	**5**	**6**	**7**	**8**	**9**	**10**		**Total**
	**1**	1											0.417 **
	**2**	−0.080	1										0.017
	**3**	0.233 **	0.025	1									0.388 **
	**4**	0.152 **	0.192 **	0.174 **	1								0.274 **
	**5**	0.056	0.190 **	0.236 **	0.269 **	1							0.362 **
	**6**	0.086	0.262 **	0.302 **	0.292 **	0.349 **	1						0.253 **
	**7**	0.259 **	0.003	0.187 **	0.133 *	0.238 **	0.249 **	1					0.363 **
	**8**	0.136 **	0.104 *	0.174 **	0.198 **	0.365 **	0.220 **	0.405 **	1				0.304 **
	**9**	0.381 **	−0.077	0.279 **	0.120 *	0.211 **	0.104 *	0.362 **	0.207 **	1			0.445 **
	**10**	0.184 **	0.115 *	0.265 **	0.262 **	0.418 **	0.362 **	0.293 **	0.330 **	0.291 **	1		0.441 **
D2—Gastronomic Innovation and Gourmet Kitchen	**Items**	**1**	**2**	**3**	**4**	**5**	**6**	**7**	**8**	**9**			**Total**
	**1**	1											0.562 **
	**2**	0.424 **	1										0.636 **
	**3**	−0.068	−0.173 **	1									0.093
	**4**	0.231 **	0.252 **	0.028	1								0.510 **
	**5**	0.346 **	0.445 **	−0.073	0.266 **	1							0.789 **
	**6**	0.279 **	0.452 **	−0.138 **	0.204 **	0.725 **	1						0.762 **
	**7**	0.283 **	0.479 **	−0.092	0.204 **	0.655 **	0.748 **	1					0.798 **
	**8**	0.354 **	0.412 **	−0.088	0.339 **	0.635 **	0.602 **	0.687 **	1				0.792 **
	**9**	0.326 **	0.388 **	−0.092	0.307 **	0.567 **	0.566 **	0.677 **	0.627 **	1			0.761 **
D3—Environment and Sustainability	**Items**	**1**	**2**	**3**	**4**	**5**	**6**	**7**	**8**	**9**	**10**	**11**	**Total**
	**1**	1											0.803 **
	**2**	0.718 **	1										0.801 **
	**3**	0.646 **	0.722 **	1									0.793 **
	**4**	0.688 **	0.702 **	0.714 **	1								0.792 **
	**5**	0.724 **	0.667 **	0.697 **	0.689 **	1							0.810 **
	**6**	−0.004	−0.003	−0.035	−0.006	0.013	1						0.244 **
	**7**	0.661 **	0.672 **	0.645 **	0.641 **	0.679 **	0.0	1					0.795 **
	**8**	0.034	−0.032	−0.026	−0.039	0.043	0.545 **	0.0	1				0.260 **
	**9**	0.386 **	0.343 **	0.424 **	0.348 **	0.389 **	0.125 *	0.435 **	0.219 **	1			0.624 **
	**10**	0.440 **	0.458 **	0.442 **	0.468 **	0.455 **	0.183 **	0.493 **	0.152 **	0.436 **	1		0.682 **
	**11**	0.637 **	0.698 **	0.678 **	0.690 **	0.652 **	0.005	0.688 **	0.061	0.557 **	0.589 **	1	0.839 **
D4—Economic and Social Aspects	**Items**	**1**	**2**	**3**	**4**	**5**	**6**						**Total**
	**1**	1											0.770 **
	**2**	0.706 **	1										0.728 **
	**3**	−0.146 **	−0.123 *	1									0.257 **
	**4**	0.467 **	0.465 **	0.113 *	1								0.768 **
	**5**	0.623 **	0.558 **	−0.002	0.544 **	1							0.801 **
	**6**	0.438 **	0.289 **	0.110 *	0.565 **	0.524 **	1						0.706 **
D5—Commercialization and Marketing	**Items**	**1**	**2**	**3**	**4**	**5**	**6**	**7**	**8**				**Total**
	**1**	1											0.519 **
	**2**	−0.201 **	1										0.249 **
	**3**	0.377 **	−0.062	1									0.631 **
	**4**	0.367 **	−0.095	0.436 **	1								0.668 **
	**5**	0.169 **	0.123 *	0.258 **	0.372 **	1							0.661 **
	**6**	0.265 **	0.054	0.351 **	0.459 **	0.437 **	1						0.703 **
	**7**	0.393 **	−0.091	0.475 **	0.549 **	0.390 **	0.551 **	1					0.701 **
	**8**	−0.038	0.278 **	0.067	−0.047	0.258 **	0.076	−0.067	1				0.360 **
D6—Nutritional Aspects	**Items**	**1**	**2**	**3**	**4**	**5**	**6**	**7**	**8**	**9**	**10**		**Total**
	**1**	1											0.059
	**2**	−0.289 **	1										0.705 **
	**3**	−0.398 **	0.777 **	1									0.689 **
	**4**	0.425 **	−0.201 **	−0.242 **	1								0.186 **
	**5**	−0.257 **	0.540 **	0.686 **	−0.160 **	1							0.746 **
	**6**	−0.113 *	0.563 **	0.590 **	−0.014	0.728 **	1						0.840 **
	**7**	−0.060	0.571 **	0.520 **	−0.007	0.600 **	0.723 **	1					0.787 **
	**8**	−0.202 **	0.595 **	0.622 **	−0.122 *	0.724 **	0.784 **	0.694 **	1				0.802 **
	**9**	0.040	0.341 **	0.320 **	0.157 **	0.385 **	0.524 **	0.450 **	0.454 **	1			0.673 **
	**10**	0.113 *	0.374 **	0.308 **	0.274 **	0.407 **	0.479 **	0.405 **	0.468 **	0.586 **	1		0.698 **
D7—Health Effects	**Items**	**1**	**2**	**3**	**4**	**5**	**6**	**7**	**8**	**9**	**10**		**Total**
	**1**	1											0.610 **
	**2**	0.408 **	1										0.703 **
	**3**	−0.045	0.031	1									0.344 **
	**4**	0.477 **	0.443 **	−0.158 **	1								0.574 **
	**5**	0.031	0.080	0.622 **	−0.132 *	1							0.434 **
	**6**	0.215 **	0.450 **	0.105 *	0.338 **	0.204 **	1						0.614 **
	**7**	0.504 **	0.574 **	0.005	0.444 **	0.115 *	0.293 **	1					0.705 **
	**8**	0.488 **	0.563 **	−0.149 **	0.502 **	−0.030	0.326 **	0.586 **	1				0.655 **
	**9**	0.294 **	0.349 **	0.240 **	0.253 **	0.310 **	0.335 **	0.394 **	0.402 **	1			0.680 **
	**10**	0.201 **	0.298 **	0.308 **	0.205 **	0.344 **	0.330 **	0.324 **	0.281 **	0.585 **	1		0.632 **

** Correlation is significant at the 0.01 level; * Correlation is significant at the 0.05 level.

**Table 2:** Internal reliability analysis of all seven subscales.

		Items											Reliability Analysis ^1^				
		1	2	3	4	5	6	7	8	9	10	11	N	αWSC	Mean	SD	Variance
	Subscale D1—Culture and Tradition																
Cronbach’s alpha considering all items		0.726	0.740	0.711	0.716	0.698	0.701	0.702	0.704	0.711	0.690		10	0.732	28.25	5.39	29.02
Cronbach’s alpha removing item 2		0.734	−	0.719	0.730	0.710	0.716	0.710	0.714	0.716	0.699		9	0.740	26.94	5.34	27.43
	Subscale D2—Gastronomic Innovation and Gourmet Kitchen																
Cronbach’s alpha considering all items		0.784	0.771	0.861	0.794	0.746	0.750	0.745	0.744	0.749			9	0.795	29.16	5.69	32.38
Cronbach’s alpha removing item 3		0.865	0.850	−	0.876	0.831	0.833	0.829	0.830	0.835			8	0.861	26.45	5.07	32.57
Cronbach’s alpha removing items 1, 3, and 4		−	0.901	−	−	0.864	0.861	0.855	0.867	0.874			6	0.890	19.25	4.72	22.24
Cronbach’s alpha removing items 1, 2, 3 and 4		−	−	−	−	0.880	0.876	0.866	0.882	0.892			5	0.901	16.01	4.13	17.09
	Subscale D3—Environment and Sustainability																
Cronbach’s alpha considering all items		0.861	0.862	0.863	0.863	0.861	0.899	0.862	0.901	0.875	0.871	0.859	11	0.882	36.09	7.54	56.86
Cronbach’s alpha removing items 6 and 8		0.915	0.914	0.915	0.915	0.915	−	0.915	−	0.932	0.927	0.912	9	0.926	30.89	7.23	52.33
Cronbach’s alpha removing items 6, 8, 9 and 10		0.922	0.920	0.921	0.920	0.921	−	0.922	−	−	−	0.920	8	0.932	27.58	6.68	44.61
	Subscale D4—Economic and Social Aspects																
Cronbach’s alpha considering all items		0.663	0.682	0.843	0.663	0.648	0.686						6	0.742	19.26	3.59	12.91
Cronbach’s alpha removing item 3		0.791	0.815	−	0.817	0.793	0.837						5	0.843	16.81	3.47	12.07
	Subscale D5—Commercialization and Marketing																
Cronbach’s alpha considering all items		0.658	0.733	0.619	0.608	0.611	0.597	0.597	0.704				8	0.675	25.12	4.26	18.14
Cronbach’s alpha removing items 2 and 8		0.791	−	0.764	0.742	0.784	0.753	0.727	−				6	0.793	20.31	3.94	15.51
	Subscale D6—Nutritional Aspects																
Cronbach’s alpha considering all items		0.856	0.767	0.770	0.834	0.760	0.749	0.755	0.752	0.771	0.769		10	0.799	30.96	5.09	25.89
Cronbach’s alpha removing items 1 and 4		−	0.891	0.890	−	0.885	0.881	0.888	0.880	0.904	0.903		8	0.903	25.93	5.11	26.14
Cronbach’s alpha removing items 1, 4 and 9		−	0.891	0.888	−	0.884	0.882	0.889	0.879	−	0.912		7	0.904	22.92	4.66	21.74
Cronbach’s alpha removing items 1, 4, 9 and 10		−	0.913	0.897	−	0.895	0.893	0.902	0.890	−	−		6	0.912	19.94	4.29	18.43
	Subscale D7—Health Effects																
Cronbach’s alpha considering all items		0.768	0.752	0.809	0.773	0.791	0.767	0.752	0.759	0.756	0.762		10	0.788	31.03	4.86	23.064
Cronbach’s alpha removing items 3 and 5		0.817	0.800	−	0.813	−	0.827	0.800	0.799	0.817	0.827		8	0.832	25.83	4.42	19.53

^1^ N = number of items considered for the analysis; αWSC = Cronbach’s alpha for the whole subscale; SD = standard deviation.

**Table 3:** Values of measure of sample adequacy (MSA).

Dimension	Item Number										
	1	2	3	4	5	6	7	8	9	10	11
D1. Culture and Tradition	0.935	0.705	0.929	0.823	0.885	0.859	0.876	0.876	0.927	0.911	
D2. Gastronomic innovation and Gourmet kitchen	0.921	0.917	0.702	0.906	0.932	0.913	0.913	0.944	0.933		
D3. Environment and Sustainability	0.950	0.935	0.966	0.959	0.965	0.668	0.952	0.659	0.908	0.908	0.942
D4. Economic and Social Aspects	0.923	0.938	0.840	0.944	0.920	0.921					
D5. Commercialization and Marketing	0.920	0.689	0.951	0.948	0.884	0.939	0.955	0.731			
D6. Nutritional Aspects	0.772	0.910	0.937	0.705	0.931	0.938	0.924	0.945	0.916	0.856	
D7. Health Effects	0.882	0.933	0.793	0.918	0.776	0.889	0.929	0.898	0.804	0.793	

**Table 4:** Validation of FA through Cronbach’s alpha calculation.

Factor	Initial			Final		Items
	N	α		N	α	
F1	10	0.937		8	0.939	D3.1, D3.2, D3.3, D3.4, D3.5, D3.7, D3.11, D4.1, D4.2, D4.4, D6.3
F2	8	0.903		6	0.912	D6.2, D6.3, D6.5, D6.6, D6.7, D6.8
F3	6	0.890		5	0.901	D2.5, D2.6, D2.7, D2.8, D2.9
F4	7	0.827	=	7	0.827	D7.1, D7.2, D7.4, D7.7, D7.8, D7.9, D7.10
F5	5	0.723		4	0.724	D7.3, D7.5, D7.9, D7.10
F6	5	0.784	=	5	0.784	D5.1, D5.3, D5.4, D5.6, D5.7
F7	5	0.705	=	5	0.705	D4.3, D5.2, D5.8, D6.1, D6.4
F8	5	0.663	=	5	0.663	D1.1, D1.7, D1.8, D1.9, D2.4
F9	5	0.654	=	5	0.654	D1.2, D1.4, D1.5, D1.6, D1.10
F10	2	0.704	=	2	0.704	D3.6, D3.8
F11	2	0.493	=	2	0.493	D4.6, D5.5
F12	2	0.339	=	2	0.339	D1.3, D4.5
F13	1	−	=	1	−	D3.9
F14	1	−	=	1	−	D2.3

## Data Availability

Data are available from the corresponding author upon request.

## Appendix A

Table A1 presents the 65 items that were included in the final version of the questionnaire approved by the ethics committee and used for statistical validation.

**Table A1:** Items that compose the questionnaire, in the final version.

Dimension	Item	Reference	Assessment
D1. Culture and Tradition	1. Entomophagy is a dietary practice that consists in the consumption of insects by humans.	[20]	knowledge
	2. Insects are considered a traditional food in my country.	[20]	perception
	3. There are thousands of species of insects that are consumed by humans in the world.	[20]	knowledge ^(1)^
	4. Consuming insects is characteristic of developing countries.	[20]	perception
	5. Insects are present in events related to religious rituals.	[49]	perception
	6. Insects are part of the gastronomic culture of most countries in the world.	[20]	perception
	7. In some countries the tradition of eating insects is decreasing because of the “Westernization” of diets.	[6]	perception
	8. Insect consumption is seasonal, so it varies according to the time of the year.	[6]	perception
	9. There are obstacles to consumers’ acceptance of edible insects in Western countries.	[6]	perception
	10. Insects can be associated with traditional festivities and celebrations.	[6]	perception
D2. Gastronomic Innovation and Gourmet Kitchen	1. Insects are considered as exotic foods.	[20]	perception
	2. Insects are traded as treats/delicacies.	[20]	perception
	3. Insects are not suitable for human consumption.	[9]	knowledge
	4. Insects are associated with taboos and food neophobia (not wanting to eat unfamiliar foods).	[6]	perception
	5. Some gourmet restaurants use edible insects in their culinary preparations.	[20]	perception
	6. Insects are present in culinary events and gastronomic shows.	[6]	perception
	7. Insects are recommended by some recognized chefs.	[6]	perception
	8. Chefs contribute to the popularization of insects into gastronomy in Western countries.	[6]	perception
	9. Culinary education favours overall liking for innovative insect based products.	[6]	perception
D3. Environment and Sustainability	1. Insects are a more sustainable alternative when compared with other sources of animal protein.	[20]	knowledge
	2. Insect production for human consumption emits much less greenhouse gases than beef production.	[20]	knowledge
	3. Insects efficiently convert organic matter into protein.	[20]	knowledge
	4. The production of insect protein uses considerably less feed than cow protein.	[20]	knowledge ^(1)^
	5. Insects are a possibility for responding to the growing world demand for protein.	[20]	knowledge
	6. The production of chicken protein requires much less water than insect protein.	[20]	knowledge ^(1)^
	7. The ecological footprint (impact) of insects is smaller when compared with other animal proteins.	[6]	knowledge
	8. The production of insect protein requires much more area than pig protein.	[20]	knowledge ^(1)^
	9. Insects are collected as a means of pest control for some cultivated crops.	[49]	knowledge
	10. The loss of biodiversity is lower with insect production compared with other animal food production.	[49]	knowledge
	11. The energy input needed for production of insect protein is lower than for the production of other proteins from animal origin.	[49]	knowledge
D4. Economic and Social Aspects	1. Insect production can contribute to increase the income of families in low income areas.	[20]	perception
	2. Insects provide protein foods at cheap prices.	[20]	perception
	3. The market for edible insects is expected to decline in the future.	[6]	perception
	4. Presently, the Asia–Pacific and Latin America areas account for more than half of the edible insects market.	[6]	perception
	5. In some countries insect farming is becoming a key factor in the fight against rural poverty.	[6]	perception
	6. The income generated from insects can be affected by market fluctuations in price derived from availability.	[49]	perception
D5. Commercialization and Marketing	1. Edible insects are difficult to find on sale in street markets.	[20]	perception
	2. Edible insects are easy to find on sale in supermarkets.	[20]	perception
	3. Edible insects are on sale only in specialized shops.	[20]	perception
	4. The level of knowledge influences the willingness to purchase insect food.	[6]	perception
	5. Price is among the motivations to consume insect foods.	[6]	perception
	6. The consumption of insects and derived foods depends on availability.	[6]	perception
	7. Personalities/influencers can lead people to consume insects.	[20]	perception
	8. Insect consumption is independent of marketing campaigns.	[6]	perception
D6. Nutritional Aspects	1. Insects have poor nutritional value.	[20]	knowledge ^(1)^
	2. Insects are a good source of energy.	[20]	knowledge
	3. Insects have high protein content.	[78]	knowledge
	4. Insect proteins are of poorer quality compared with other animal species.	[20]	knowledge ^(1)^
	5. Insects provide essential amino acids necessary for humans.	[78]	knowledge
	6. Insects contain group B vitamins.	[20]	knowledge
	7. Insects contain dietary fibre.	[78]	knowledge
	8. Insects contain minerals of nutritional interest, such as calcium, iron and magnesium.	[20]	knowledge
	9. Insects contain fat, including unsaturated fatty acids.	[20,49]	knowledge
	10. Insects contain anti-nutrients, such as oxalates and phytic acid.	[72]	knowledge
D7. Health Effects	1. There are appropriate regulations to guarantee the food safety of edible insects.	[20]	knowledge
	2. Insects are used by some people in traditional medicine.	[20]	perception
	3. Eating insects poses a substantial risk to human health.	[9]	perception
	4. Industrially processed insect products are hygienic and safe.	[9]	perception
	5. Insects and insect-based foods are often infected by pathogens and parasites.	[80]	perception
	6. Insects collected from the wild may be contaminated with pesticide residues.	[81]	perception
	7. In certain countries, insects are approved officially for therapeutic treatment.	[49]	perception
	8. Insects contain bioactive compounds beneficial to human health.	[20]	knowledge
	9. Insects are potential sources of allergens.	[81]	knowledge
	10. Aflatoxins, which are carcinogens, can be present in insects.	[81]	knowledge

^(1)^ False statement.

## Appendix B

Table A2 presents the loadings of the different items into the fourteen factors extracted through the factor analysis.

**Table A2:** Results (loadings) of the FA solution with extraction by PCA with varimax rotation (loadings lower than 0.4 were excluded).

	F1	F2	F3	F4	F5	F6	F7	F8	F9	F10	F11	F12	F13	F14
**D1.1**								0.418						
**D1.2**									0.546					
**D1.3**												−0.461		
**D1.4**									0.587					
**D1.5**									0.602					
**D1.6**									0.655					
**D1.7**								0.596						
**D1.8**								0.449						
**D1.9**								0.569						
**D1.10**									0.490					
**D2.1**														
**D2.2**			0.476											
**D2.3**														0.809
**D2.4**								0.556						
**D2.5**			0.778											
**D2.6**			0.816											
**D2.7**			0.809											
**D2.8**			0.696											
**D2.9**			0.646											
**D3.1**	0.730													
**D3.2**	0.798													
**D3.3**	0.707													
**D3.4**	0.771													
**D3.5**	0.702													
**D3.6**										0.698				
**D3.7**	0.742													
**D3.8**										0.795				
**D3.9**	0.437												0.547	
**D3.10**	0.627													
**D3.11**	0.813													
**D4.1**	0.502													
**D4.2**	0.576													
**D4.3**					0.435		0.400							
**D4.4**	0.488													
**D4.5**												0.490		
**D4.6**											0.406			
**D5.1**						0.548								
**D5.2**							0.669							
**D5.3**						0.619								
**D5.4**						0.550								
**D5.5**											0.747			
**D5.6**						0.533								
**D5.7**						0.561								
**D5.8**							0.536							
**D6.1**							0.585							
**D6.2**		0.595												
**D6.3**	0.406	0.584												
**D6.4**							0.574							
**D6.5**		0.697												
**D6.6**		0.777												
**D6.7**		0.698												
**D6.8**		0.785												
**D6.9**		0.596												
**D6.10**		0.646												
**D7.1**				0.701										
**D7.2**				0.603										
**D7.3**					0.780									
**D7.4**				0.569										
**D7.5**					0.790									
**D7.6**														
**D7.7**				0.684										
**D7.8**				0.693										
**D7.9**				0.490	0.462									
**D7.10**				0.431	0.482									

## References

[1] Yamane T., Kaneko S. (2022). The Sustainable Development Goals as New Business Norms: A Survey Experiment on Stakeholder Preferences. Ecol. Econ..

[2] Viana C.M., Freire D., Abrantes P., Rocha J., Pereira P. (2022). Agricultural Land Systems Importance for Supporting Food Security and Sustainable Development Goals: A Systematic Review. Sci. Total Environ..

[3] van Huis A., Oonincx D.G.A.B. (2017). The Environmental Sustainability of Insects as Food and Feed. A Review. Agron. Sustain. Dev..

[4] Herrero M., Henderson B., Havlík P., Thornton P.K., Conant R.T., Smith P., Wirsenius S., Hristov A.N., Gerber P., Gill M. (2016). Greenhouse Gas Mitigation Potentials in the Livestock Sector. Nat. Clim. Chang..

[5] Thomaz E.L., Nunes D.D., Watanabe M. (2020). Effects of Tropical Forest Conversion on Soil and Aquatic Systems in Southwestern Brazilian Amazonia: A Synthesis. Environ. Res..

[6] Guiné R.P.F., Correia P., Coelho C., Costa C.A. (2021). The Role of Edible Insects to Mitigate Challenges for Sustainability. Open Agric..

[7] Lange K.W., Nakamura Y. (2021). Edible Insects as Future Food: Chances and Challenges. J. Future Foods.

[8] Guiné R.P.F., Florença S.G., Anjos O., Correia P.M.R., Ferreira B.M., Costa C.A. (2021). An Insight into the Level of Information about Sustainability of Edible Insects in a Traditionally Non-Insect-Eating Country: Exploratory Study. Sustainability.

[9] Orsi L., Voege L.L., Stranieri S. (2019). Eating Edible Insects as Sustainable Food? Exploring the Determinants of Consumer Acceptance in Germany. Food Res. Int..

[10] Ordoñez-Araque R., Egas-Montenegro E. (2021). Edible Insects: A Food Alternative for the Sustainable Development of the Planet. Int. J. Gastron. Food Sci..

[11] da Silva Lucas A.J., de Oliveira L.M., da Rocha M., Prentice C. (2020). Edible Insects: An Alternative of Nutritional, Functional and Bioactive Compounds. Food Chem..

[12] Castro-López C., Santiago-López L., Vallejo-Cordoba B., González-Córdova A.F., Liceaga A.M., García H.S., Hernández-Mendoza A. (2020). An Insight to Fermented Edible Insects: A Global Perspective and Prospective. Food Res. Int..

[13] del Hierro J.N., Gutiérrez-Docio A., Otero P., Reglero G., Martin D. (2020). Characterization, Antioxidant Activity, and Inhibitory Effect on Pancreatic Lipase of Extracts from the Edible Insects Acheta Domesticus and Tenebrio Molitor. Food Chem..

[14] Nongonierma A.B., FitzGerald R.J. (2017). Unlocking the Biological Potential of Proteins from Edible Insects through Enzymatic Hydrolysis: A Review. Innov. Food Sci. Emerg. Technol..

[15] Patel S., Suleria H.A.R., Rauf A. (2019). Edible Insects as Innovative Foods: Nutritional and Functional Assessments. Trends Food Sci. Technol..

[16] Rumpold B.A., Schlüter O.K. (2013). Nutritional Composition and Safety Aspects of Edible Insects. Mol. Nutr. Food Res..

[17] Sun-Waterhouse D., Waterhouse G.I.N., You L., Zhang J., Liu Y., Ma L., Gao J., Dong Y. (2016). Transforming Insect Biomass into Consumer Wellness Foods: A Review. Food Res. Int..

[18] Lange K., Nakamura Y. (2021). Edible Insects as a Source of Food Bioactives and Their Potential Health Effects. J. Food Bioact..

[19] Dion-Poulin A., Turcotte M., Lee-Blouin S., Perreault V., Provencher V., Doyen A., Turgeon S.L. (2021). Acceptability of Insect Ingredients by Innovative Student Chefs: An Exploratory Study. Int. J. Gastron. Food Sci..

[20] Florença S.G., Correia P.M.R., Costa C.A., Guiné R.P.F. (2021). Edible Insects: Preliminary Study about Perceptions, Attitudes, and Knowledge on a Sample of Portuguese Citizens. Foods.

[21] Ferrão A.C., Guine R.P.F., Correia P.M., Ferreira M., Duarte J., Lima J. (2019). Development of A Questionnaire To Assess People’s Food Choices Determinants. Curr. Nutr. Food Sci..

[22] Hill M.M., Hill A. (2009). Investigação Por Questionário.

[23] Likert R. (1932). A Technique for the Measurement of Attitudes. Arch. Psychol..

[24] da Silva A.M., Barroca M.J., Guiné R.P.F. (2021). Knowledge and Consumption Habits Related with White Crowberries (*Corema album* L.). Appl. Sci..

[25] Wongprawmas R., Mora C., Pellegrini N., Guiné R.P.F., Carini E., Sogari G., Vittadini E. (2021). Food Choice Determinants and Perceptions of a Healthy Diet among Italian Consumers. Foods.

[26] Macena M.W., Carvalho R., Cruz-Lopes L.P., Guiné R.P.F. (2021). Plastic Food Packaging: Perceptions and Attitudes of Portuguese Consumers about Environmental Impact and Recycling. Sustainability.

[27] Georgescu M., Tarcea M., Hardmas R., Seni G., Teodorescu C., Szasz S., Guiné R., Abram Z. (2020). Romanian Population Perception about Food Risk Behavior Starting from Their Social and Cultural Profile. Bull. Univ. Agric. Sci. Vet. Med. Cluj-Napoca. Food Sci. Technol..

[28] Marôco J. (2012). Análise Estatística Com o SPSS Statistics.

[29] Pestana M.H., Gageiro J.N. (2014). Análise de Dados Para Ciências Sociais–A Complementaridade Do SPSS.

[30] Stevens J.P. (2009). Applied Multivariate Statistics for the Social Sciences.

[31] Broen M.P.G., Moonen A.J.H., Kuijf M.L., Dujardin K., Marsh L., Richard I.H., Starkstein S.E., Martinez–Martin P., Leentjens A.F.G. (2015). Factor Analysis of the Hamilton Depression Rating Scale in Parkinson’s Disease. Parkinsonism Relat. Disord..

[32] Hellstrom W.J.G., Feldman R., Rosen R.C., Smith T., Kaufman G., Tursi J. (2013). Bother and Distress Associated with Peyronie’s Disease: Validation of the Peyronie’s Disease Questionnaire. J. Urol..

[33] Guiné R.P.F., Correia P., Leal M., Rumbak I., Barić I.C., Komes D., Satalić Z., Sarić M.M., Tarcea M., Fazakas Z. (2020). Cluster Analysis to the Factors Related to Information about Food Fibers: A Multinational Study. Open Agric..

[34] Rohm A.J., Swaminathan V. (2004). A Typology of Online Shoppers Based on Shopping Motivations. J. Bus. Res..

[35] Tanaka K., Akechi T., Okuyama T., Nishiwaki Y., Uchitomi Y. (2000). Development and Validation of the Cancer Dyspnoea Scale: A Multidimensional, Brief, Self-Rating Scale. Br. J. Cancer.

[36] Kaiser H.F., Rice J. (1974). Little Jiffy, Mark Iv. Educ. Psychol. Meas..

[37] Hair J.F.H., Black W.C., Babin B.J., Anderson R.E. (2009). Multivariate Data Analysis.

[38] Maroco J., Garcia-Marques T. (2006). Qual a fiabilidade do alfa de Cronbach? Questões antigas e soluções modernas?. Laboratório De Psicol..

[39] Davis F.B. (1964). Educational Measurements Their Interpretation.

[40] Müller A., Evans J., Payne C.L.R., Roberts R. (2016). Entomophagy and Power. J. Insects Food Feed..

[41] Tabassum-Abbasi, Abbasi T., Abbasi S.A. (2016). Reducing the Global Environmental Impact of Livestock Production: The Minilivestock Option. J. Clean. Prod..

[42] Gjerris M., Gamborg C., Röcklinsberg H. (2016). Ethical Aspects of Insect Production for Food and Feed. J. Insects Food Feed..

[43] Vázquez A.P., Trinidad D.A.L., Merino F.C.G. (2018). Desafíos y propuestas para lograr la seguridad alimentaria hacia el año 2050. Rev. Mex. De Cienc. Agrícolas.

[44] House J. (2018). Insects as Food in the Netherlands: Production Networks and the Geographies of Edibility. Geoforum.

[45] Nowak V., Persijn D., Rittenschober D., Charrondiere U.R. (2016). Review of Food Composition Data for Edible Insects. Food Chem..

[46] Youssef J., Spence C. (2021). Introducing Diners to the Range of Experiences in Creative Mexican Cuisine, Including the Consumption of Insects. Int. J. Gastron. Food Sci..

[47] Zahid M., Rahman H.U., Ullah Z., Muhammad A. (2021). Sustainability and Branchless Banking: The Development and Validation of a Distinct Measurement Scale. Technol. Soc..

[48] Pliner P., Hobden K. (1992). Development of a Scale to Measure the Trait of Food Neophobia in Humans. Appetite.

[49] Gahukar R.T. (2020). Edible Insects Collected from Forests for Family Livelihood and Wellness of Rural Communities: A Review. Glob. Food Secur..

[50] Ghosh S., Jung C., Meyer-Rochow V.B., Halloran A., Flore R., Vantomme P., Roos N. (2018). What Governs Selection and Acceptance of Edible Insect Species?. Edible Insects in Sustainable Food Systems.

[51] Megu K., Chakravorty J., Meyer-Rochow V.B., Halloran A., Flore R., Vantomme P., Roos N. (2018). An Ethnographic Account of the Role of Edible Insects in the Adi Tribe of Arunachal Pradesh, North-East India. Edible Insects in Sustainable Food Systems.

[52] Séré A., Bougma A., Ouilly J.T., Traoré M., Sangaré H., Lykke A.M., Ouédraogo A., Gnankiné O., Bassolé I.H.N. (2018). Traditional Knowledge Regarding Edible Insects in Burkina Faso. J. Ethnobiol. Ethnomedicine.

[53] Barrea L., Muscogiuri G., Di Somma C., Tramontano G., De Luca V., Illario M., Colao A., Savastano S. (2019). Association between Mediterranean Diet and Hand Grip Strength in Older Adult Women. Clin. Nutr..

[54] Park S.-Y., Murphy S.P., Wilkens L.R., Yamamoto J.F., Sharma S., Hankin J.H., Henderson B.E., Kolonel L.N. (2005). Dietary Patterns Using the Food Guide Pyramid Groups Are Associated with Sociodemographic and Lifestyle Factors: The Multiethnic Cohort Study. J. Nutr..

[55] Sousa B. (2018). Mediterranean Diet, a Way to Reduce Atherosclerosis: The Adherence in a Portuguese Sample. Atherosclerosis.

[56] Hwang J., Choe J.Y. (2020). How to Enhance the Image of Edible Insect Restaurants: Focusing on Perceived Risk Theory. Int. J. Hosp. Manag..

[57] Lambert H., Elwin A., D’Cruze N. (2021). Wouldn’t Hurt a Fly? A Review of Insect Cognition and Sentience in Relation to Their Use as Food and Feed. Appl. Anim. Behav. Sci..

[58] Tanga C.M., Egonyu J.P., Beesigamukama D., Niassy S., Emily K., Magara H.J., Omuse E.R., Subramanian S., Ekesi S. (2021). Edible Insect Farming as an Emerging and Profitable Enterprise in East Africa. Curr. Opin. Insect Sci..

[59] Ali L., Ali F. (2022). Perceived Risks Related to Unconventional Restaurants: A Perspective from Edible Insects and Live Seafood Restaurants. Food Control.

[60] Murefu T.R., Macheka L., Musundire R., Manditsera F.A. (2019). Safety of Wild Harvested and Reared Edible Insects: A Review. Food Control.

[61] Baiano A. (2020). Edible Insects: An Overview on Nutritional Characteristics, Safety, Farming, Production Technologies, Regulatory Framework, and Socio-Economic and Ethical Implications. Trends Food Sci. Technol..

[62] Yates-Doerr E. (2015). The World in a Box? Food Security, Edible Insects, and "One World, One Health" Collaboration. Soc. Sci. Med..

[63] European Commission Approval of Second Insect as a Novel Food. https://ec.europa.eu/food/safety/novel-food/authorisations/approval-second-insect-novel-food_en.

[64] Sarić M.M., Jakšić K., Čulin J., Guiné R.P.F. (2020). Environmental and Political Determinants of Food Choices: A Preliminary Study in a Croatian Sample. Environments.

[65] Guiné R.P.F., Bartkiene E., Florença S.G., Djekić I., Bizjak M.Č., Tarcea M., Leal M., Ferreira V., Rumbak I., Orfanos P. (2021). Environmental Issues as Drivers for Food Choice: Study from a Multinational Framework. Sustainability.

[66] Bombe K. (2019). Edible Insects Market Worth $7.96 Billion by 2030.

[67] Goldstein D. (2018). Edible Insect Market Growth.

[68] Jornal de Negócios Snacks, Farinha e Barras Proteicas. Continente já Vende Alimentos à Base de Insetos [Snacks, Flour and Protein Bars. Continente Already Sells Insect-Based Food]. https://www.jornaldenegocios.pt/empresas/detalhe/snacks-farinha-e-barras-proteicas-continente-ja-vende-alimentos-a-base-de-insetos.

[69] DGAV (2021). Insetos–Colocação No Mercado Ao Abrigo de Medidas Transitórias [Insects–Placing on the Market under Transitional Measures] (In Portuguese).

[70] Agbidye F., Ofuya T., Akindele S. (2009). Some Edible Insect Species Consumed by the People of Benue State, Nigeria. Pak. J. Nutr..

[71] Ojha S., Bekhit A.E.-D., Grune T., Schlüter O.K. (2021). Bioavailability of Nutrients from Edible Insects. Curr. Opin. Food Sci..

[72] Kunatsa Y., Chidewe C., Zvidzai C.J. (2020). Phytochemical and Anti-Nutrient Composite from Selected Marginalized Zimbabwean Edible Insects and Vegetables. J. Agric. Food Res..

[73] Chakravorty J., Ghosh S., Megu K., Jung C., Meyer-Rochow V.B. (2016). Nutritional and Anti-Nutritional Composition of Oecophylla Smaragdina (Hymenoptera: Formicidae) and *Odontotermes* Sp. (Isoptera: Termitidae): Two Preferred Edible Insects of Arunachal Pradesh, India. J. Asia-Pac. Entomol..

[74] Baker M.A., Shin J.T., Kim Y.W. (2016). An Exploration and Investigation of Edible Insect Consumption: The Impacts of Image and Description on Risk Perceptions and Purchase Intent. Psychol. Mark..

[75] Niva M., Vainio A. (2021). Towards More Environmentally Sustainable Diets? Changes in the Consumption of Beef and Plant- and Insect-Based Protein Products in Consumer Groups in Finland. Meat Sci..

[76] La Barbera F., Verneau F., Videbæk P.N., Amato M., Grunert K.G. (2020). A Self-Report Measure of Attitudes toward the Eating of Insects: Construction and Validation of the Entomophagy Attitude Questionnaire. Food Qual. Prefer..

[77] Lammers P., Ullmann L.M., Fiebelkorn F. (2019). Acceptance of Insects as Food in Germany: Is It about Sensation Seeking, Sustainability Consciousness, or Food Disgust?. Food Qual. Prefer..

[78] Dupont J., Fiebelkorn F. (2020). Attitudes and Acceptance of Young People toward the Consumption of Insects and Cultured Meat in Germany. Food Qual. Prefer..

[79] Chow C.-Y., Riantiningtyas R.R., Sørensen H., Frøst M.B. (2021). School Children Cooking and Eating Insects as Part of a Teaching Program–Effects of Cooking, Insect Type, Tasting Order and Food Neophobia on Hedonic Response. Food Qual. Prefer..

[80] Gałęcki R., Sokół R. (2019). A Parasitological Evaluation of Edible Insects and Their Role in the Transmission of Parasitic Diseases to Humans and Animals. PLoS ONE.

[81] Imathiu S. (2020). Benefits and Food Safety Concerns Associated with Consumption of Edible Insects. NFS J..

